# The Process and Strategy for Developing Selective Histone Deacetylase 3 Inhibitors

**DOI:** 10.3390/molecules23030551

**Published:** 2018-03-02

**Authors:** Fangyuan Cao, Martijn R. H. Zwinderman, Frank J. Dekker

**Affiliations:** Chemical and Pharmaceutical Biology, Groningen Research Institute of Pharmacy, University of Groningen, 9713AV Groningen, The Netherlands; f.cao@rug.nl (F.C.); r.h.zwinderman@rug.nl (M.R.H.Z.)

**Keywords:** histone deacetylase 3 (HDAC3), selective, drug discovery, inhibitor, isoenzyme

## Abstract

Histone deacetylases (HDACs) are epigenetic drug targets that have gained major scientific attention. Inhibition of these important regulatory enzymes is used to treat cancer, and has the potential to treat a host of other diseases. However, currently marketed HDAC inhibitors lack selectivity for the various HDAC isoenzymes. Several studies have shown that HDAC3, in particular, plays an important role in inflammation and degenerative neurological diseases, but the development of selective HDAC3 inhibitors has been challenging. This review provides an up-to-date overview of selective HDAC3 inhibitors, and aims to support the development of novel HDAC3 inhibitors in the future.

## 1. Introduction

During the last decades, epigenetics has been established as a crucial factor in cancer and inflammatory diseases [1,2]. Epigenetics encompasses all inheritable changes in gene expression of eukaryotic cells without changes in the genetic code. This process is carried out by a range of mechanisms, an important one being the association of DNA with histone and non-histone proteins, resulting in the formation of chromatin. When DNA interacts tightly with these proteins, gene transcription is reduced. The histone proteins are subjected to posttranslational modifications, including methylation, acetylation, phosphorylation, and ubiquitination, which confer epigenetic regulation of gene transcription [3,4]. Acetylation of histones has become a widely studied process in the last decade, as it has been linked to various diseases, such as cancer and inflammation [1,3]. Histone acetylation is under the control of histone acetyltransferases (HATs) and histone deacetylases (HDACs) that acetylate and deacetylate the N-terminal lysine residues of histones, respectively [5]. HATs transfer acetyl groups onto the lysine residues of histones, causing them to lose their positively charged character used for the association with negatively charged DNA. HDACs have the opposite effect on histone lysine residues, and deacetylation leads to a more condensed chromatin structure, making it less accessible for the transcription machinery [6,7]. Besides histones, HDACs also play an important role in the deacetylation of non-histone proteins, such as α-tubulin, transcription factors, and nuclear transport proteins, and are therefore involved in several signal transduction pathways [8]. HDACs have been an important drug target to treat disorders where deacetylation is distorted, such as cancer, but they are also emerging as a target for other diseases, such as neurological disorders and inflammatory, cardiac, and pulmonary diseases [9]. In cancer, HDAC inhibitors induce apoptosis of tumor cells by interfering with cell growth [10,11,12] and differentiation [10,13]. It is also reported that HDAC inhibitors work synergistically in cancer therapies for B-cell lymphoma 2 (BCL2) [14] and therapies targeting TNF-related apoptosis-inducing ligand (TRAIL) [15] and tyrosine kinases [16]. Besides, HDAC inhibitors enhance sensitivity of cells to DNA damage [17], which indicates that inhibition of HDACs may play an important role in DNA repair pathways in human cells. However, a remaining challenge is to develop selective inhibitors for the different HDAC isoenzymes, and to unravel the functions of these HDAC isoenzymes in specific disease models.

HDAC3 is one of the HDAC isoenzymes for which important roles have been described in cancer, inflammation, and degenerative neurological diseases [18,19,20]. Therefore, development and application of selective HDAC3 inhibitors is expected to enable drug discovery. To support the development of novel HDAC3 inhibitors, this paper reviews the currently available HDAC3 selective inhibitors, and discusses new directions in the development of selective HDAC3 inhibitors. 

## 2. Results

### 2.1. HDAC Subtypes

To date, 18 HDAC subtypes are known, which are divided into two families and four classes, based on sequence similarity and cofactor dependency. The first family consists of HDAC classes I, II, and IV, and comprises the “classical” zinc-dependent HDACs, while class III consists of the NAD^+^-dependent sirtuin (SIRT1-7) family [8,9]. Generally, class I HDACs, which are HDAC1, 2, 3, and 8, are located primarily in the nucleus. Class II HDACs, which comprises class IIA, including HDAC4, 5, 7, and 9, and class IIB, including HDAC6 and HDAC10, also have major cytoplasmic functions. HDAC11 is the only class IV HDAC, and together with HDAC10, is the most poorly understood HDAC subtype. The class III sirtuins contain both mono-ADP-ribosyltransferase and histone deacetylase activity, and are located in the nucleus, the mitochondria, or the cytoplasm, depending on the isoform [9]. 

### 2.2. The Promise of Selective HDAC3 Inhibition

**Figure 1:**
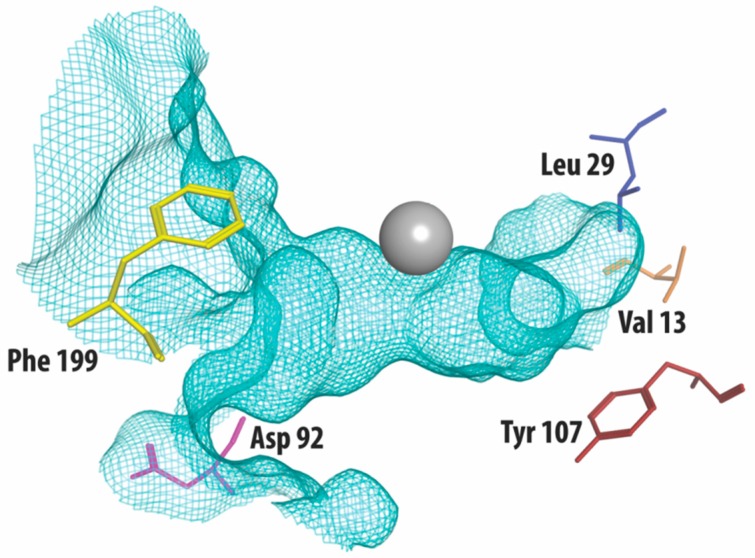
The crystal structure of HDAC3 (PDB code: 4A69, mesh indicates the catalytic site surface) showing only the amino acid residues that differ from HDAC1 and HDAC2 (presented as stick structures). The zinc required for catalysis is shown in grey.

Class I HDACs are currently the most studied of all HDACs. Apart from HDAC8, class I HDACs have a similar structure, especially considering the area near the substrate-binding site [21]. Figure 1 shows part of the crystal structure of HDAC3 that makes up the active site with the catalytic zinc ion in grey. A structural alignment of HDAC3 with HDAC1 and 2 reveals five differences [22,23], shown in Table 1. In the outer rim of its cavity, HDAC3 has an aspartate residue in position 92, instead of glutamate, found in both HDAC1 and 2. Furthermore, HDAC3 has an additional phenylalanine in position 199, whereas this is not present in HDAC 1 and 2. Instead, structural alignment reveals that a tyrosine residue occupies this location in HDAC 1 and 2. Most notably, the tyrosine in position 107 in HDAC 3 is a serine in HDAC 1 and 2, which is a difference that has been used to rationalize the identification of HDAC1/2 selective inhibitors [24]. In contrast to the serines in HDAC 1 and 2, the tyrosine 107 in HDAC3 provides steric hindrance for binding to the foot pocket, thus precluding binding of inhibitors with larger functional groups in this position [25,26]. Even deeper in the foot pocket in HDAC 1, 2, and 3, there are different hydrophobic amino acids at positions 29 and 13, thus providing small structural differences that could be employed for the development of selective binders [26].

It is known that class I HDACs are overexpressed in various human cancers, including cancer of the stomach, esophagus, colon, breast, ovaries, lung, pancreas, and thyroid [27]. Inhibiting the class I HDACs might therefore be useful for the treatment of a wide variety of human cancers. More recently, HDAC inhibitors are under investigation for controlling cancer stem cells (CSC) in tumors, which are responsible for invasiveness, drug resistance, and relapse of tumor growth [28]. It is also reported that inhibition of HDAC3 could induce cell autophagy in human glioma cells [29], and apoptosis in cholangiocarcinoma [30]. Besides their utilization as targets in cancer therapy, class I HDACs have gained considerable attention as targets in the search for treatments for degenerative neurological diseases, such as Alzheimer’s and Huntington’s disease [20,31,32,33,34], chronic inflammatory diseases, like asthma and COPD [35,36,37,38], viral infections [39], especially for human immunodeficiency virus (HIV) [40,41,42], and diabetes [22,43,44,45]. HDAC3, in particular, is an interesting target in Alzheimer’s disease [31,46], since it is reported that HDAC3 plays an important role in maintaining long-term memory for object location [47]. Moreover, selective HDAC3 inhibitors can impede Huntington’s disease-related gene expansion, and thereby protect against cognitive decline [33]. The benefits for Huntington’s disease may also be related to macrophage migration inhibitory factor (Mif), which could be downregulated by selective HDAC3 inhibitor in mice [34]. With respect to the inflammatory lung diseases asthma and COPD, HDAC3 is reportedly an important regulator of inflammation [19,48]. HDAC3 selective inhibitors can, for instance, increase the acetylation status of the NF-κB pathway [49]. In precision-cut lung slices (PCLS), a selective HDAC3 inhibitor is shown to increase the expression of IL-10, an important anti-inflammatory cytokine, and decrease the gene expression of pro-inflammatory cytokines by attenuating NF-κB p65 transcriptional activity [48]. HDAC3 also contributes to the repression of HIV-1 long terminal repeat (LTR) expression [42], and a selective HDAC3 inhibitor activates HIV-1 transcription in the 2D10 cell line, inducing outgrowth of HIV-1 from cells [40]. Moreover, knocking down HDAC3 protects pancreatic β-cell from cytokine-induced apoptosis, and could restore glucose-stimulated insulin secretion (GSIS) [22,44]. Treatment with a selective HDAC3 inhibitor also reduces hyperglycemia and increases insulin secretion in type-2 diabetes in mice [43], which indicates that HDAC3 may be a potential target for the therapy of diabetes.

### 2.3. Available HDAC3 Selective Inhibitors

**Figure 2:**
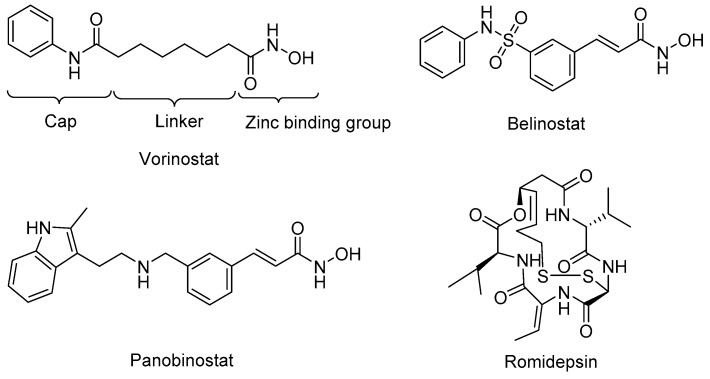
Chemical structures of HDAC inhibitors that are FDAapproved for use in cancer therapy.

Many HDAC inhibitors have been developed, including four U.S. Food and Drug Administration (FDA) approved anticancer drugs; vorinostat (SAHA) [50], belinostat [51], panobinostat [52], and romidepsin [53] (Figure 2). Most of the currently available small molecule HDAC inhibitors share the same zinc-binding group (ZBG), which binds the zinc ion that is located in the active site of class I HDACs. To mimic the lysine alkyl side chain, most HDAC inhibitors have a linker that goes into the hydrophobic tunnel that connects the ZBG to a cap group that is on the edge of the active site (Figure 2). For the ZBG, the most widely known groups are hydroxamic acids and *o*-aminoanilides. Molecules with hydroxamic acids groups, such as vorinostat, belinostat, and panobinostat, have the tendency to show non-selective inhibition towards HDAC1, 2, and 3. The *o*-aminoanilides mainly target those isoforms, and class I selective inhibitors have been made with this ZBG. The prime example of an inhibitor with a *o*-aminoanilide group is entinostat (MS-275) (Table 2), which inhibits HDAC1-3 with IC_50_ values of 0.19 μM, 0.41 μM, and 0.95 μM, respectively [19]. Further development of inhibitors in the *o*-aminoanilide class led to the development of analogues with selective HDAC3 inhibition. A well-known example is inhibitor RGFP966 with an IC_50_ value of 0.08 μM for HDAC3, and a lack of potency for other HDACs at concentrations up to 15 μM [54]. This compound is widely used as a molecular tool to study the role of HDAC3. Another selective inhibitor PD106 was developed as a drug candidate to target Friedreich’s ataxia. This compound has a K_i_ value of 14 nM for HDAC3, which was 10 times lower than the K_i_ for HDAC1 [55,56], while its analog, RGFP109, which is also used as a promising treatment for Friedreich’s ataxia, shows a better potency for HDAC3 with the Ki value of 5 nM, and 32 nM for HDAC1 [57]. BRD3308 is also known to be a HDAC3 selective inhibitor with the IC_50_ value of 0.064 μM for HDAC3, but 1.08 μM and 1.15 μM for HDAC1 and 2, respectively [22]. This compound also shows potency for the therapy of HIV infection [40] and diabetes [43,45]. 

Given the potential to target class I HDACs by inhibitors with a *o*-aminoanilide scaffold, many derivatives around this scaffold were synthesized (Table 3). For example, compound 1 was synthesized through on-resin solid-phase peptide synthesis (SPPS), to provide a flexible linker with amide bonds. This compound provides IC_50_s for HDAC1 and HDAC3, of 83.9 μM and 4.3 μM, respectively [58]. Chen et al. have reported compound 2 as a selective HDAC3 inhibitor, with an IC_50_ of 0.12 μM for HDAC3, and no inhibition of other HDACs at concentrations up to 30 μM [59]. By using click chemistry-based combinatorial fragment assembly, Suzuki et al. have screened a series of 504 candidates to obtain compound 3 and 4 as HDAC3 selective inhibitors, with IC_50_s of 0.24 μM and 0.26 μM, respectively [49]. The docking mode of compound 3 with HDAC3 showed that the NH_2_ and CO moieties of the *o*-aminoanilide group bind to the zinc ion, and also form two hydrogen bonds with His134 and Gly143. The phenyltriazole group of the compound fits in the hydrophobic tunnel through hydrophobic interactions, with another hydrophobic interaction between the thiophene ring in the cap region, and Pro23 and Phe144 [49]. In another example, chiral compound 5 has an IC_50_ of 12 nM for HDAC3, with a 7-fold higher concentration needed for HDAC2 inhibition, and about 1000-fold higher for HDAC1. Molecular modeling of inhibitor 5 with HDAC3 confirmed that the *o*-aminoanilide binds the zinc ion, and that the phenyl ring formed a hydrophobic interaction between the Phe144 and Phe200 residues in the hydrophobic tunnel [60]. Interestingly, another hydrophobic interaction was formed in the cap region with Phe199 [60]. Yu et al. have reported that compound 6 and 7 are two promising HDAC3 selective inhibitors, with 0.35 μM and 0.2 μM potency [61]. These two compounds have IC_50_ values for HDAC1 and HDAC2 higher than 10 μM, and compound 6 showed in vitro efficacy in suppressing the cancer stem cell subpopulation of triple-negative breast cancer by downregulating β-catenin [61]. McClure et al. synthesized compound 8 as a selective HDAC3 inhibitor with IC_50_ of 1.2 μM, 1.5 μM, and 0.08 μM for HDAC1, 2, and 3, respectively [62]. More interestingly, compound 9, which has another fluorine atom in the meta position of the amine of *o*-aminoanilide, shows a decrease in potency for HDACs, but a better selectivity profile for HDAC3 [62]. Furthermore, the compounds bearing a fluorine atom in the ortho position of the amine or amide lose their potency for HDACs [62], indicating that the substitution pattern of *o*-aminoanilides contributes to their selectivity and potency for HDACs.

### 2.4. Strategies for Developing Selective HDAC3 Inhibitors

Many HDAC inhibitors were synthesized, and their isoenzyme selectivity profiles were investigated. However, fully isoenzyme-selective HDAC inhibitors are rare. Most of the reported selective inhibitors still inhibit other HDACs to some extent, due to the high structural similarity in the HDAC enzyme family. The HDAC3 inhibitors mentioned in this paper are all *o*-aminoanilide derivatives, and the selectivity of most of these compounds is evaluated by measuring the IC_50_s. However, for this group of compounds, this might not be the best choice [55,64]. The classical competitive inhibitors for HDACs with a hydroxamic acid as zinc binding group bind to these enzymes with rapid-on/rapid-off kinetics. By contrast, the *o*-aminoanilides inhibit HDACs through a slow-on/slow-off kinetic mechanism [55]. Consequently, the K_i_ value of the *o*-aminoanilides should be calculated from directly measured k_on_ and k_off_ values of the inhibitors, rather than through IC_50_ values. This would provide a better indication of the selectivity profile of HDAC inhibition for this compound class [25,55,56]. 

**Figure 3:**
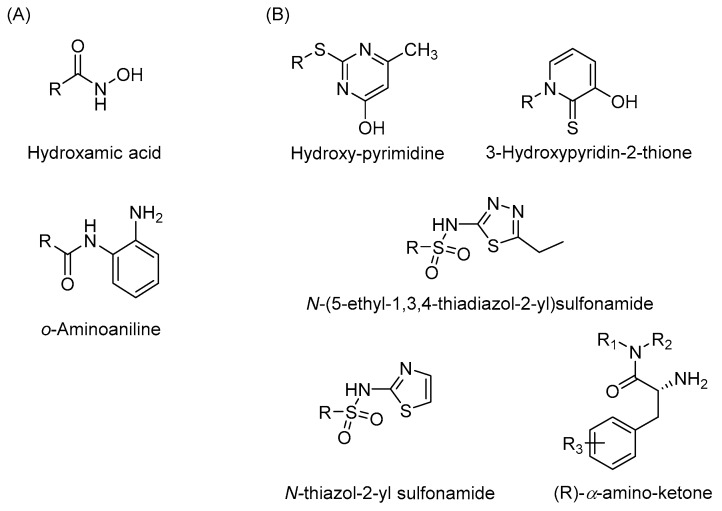
The zinc binding group of HDAC inhibitors. (**A**) The most studied zinc binding groups of HDAC inhibitors. (**B**) Novel zinc binding groups of HDAC inhibitors.

Development of novel zinc binding groups as scaffolds to develop selective HDAC isoenzyme inhibitors is highly needed. As demonstrated in previous studies, the addition of a fluorine atom on the *o*-aminoanilide increases the selectivity and potency to HDAC3 [61]. This demonstrates that small structural changes in the zinc binding group greatly influence HDAC isoenzyme selectivity. In addition, several studies report the application of novel zinc binding groups including hydroxypyrimidine derivatives [65], (*R*)-α-amino-ketones [66], 3-hydroxypyridin-2-thione derivatives [67], *N*-(5-ethyl-1,3,4-thiadiazol-2-yl)sulfonamides, and *N*-thiazol-2-yl sulfonamides [68] (Figure 3). Further development of these scaffolds might provide HDAC inhibitors with completely new isoenzyme selectivities. We also note that currently, the linker and cap groups of HDAC inhibitors mainly consist of rigid aromatic functionalities. It would be interesting to explore novel chemical spaces for the linker and cap groups by application of non-aromatic functionalities that are rich in sp^3^-hybridized atoms. Altogether, we envision that exploring novel chemical space around the zinc binding group and in the linker and cap group of HDAC inhibitors has great potential to come up with novel isoenzyme selective inhibitors. This will enable targeting of a diverse array of disease conditions in which these isoenzymes play distinct roles. 

## 3. Conclusions

For over two decades, HDACs have been considered as attractive targets in drug discovery. A large number of HDAC inhibitors have been developed and patented, and a substantial amount has entered clinical trials. Moreover, the FDA has approved four HDAC inhibitors for the treatment of cancer patients in the United States. However, all of the HDAC inhibitors in clinical use are pan-HDAC inhibitors. To improve the current generation of clinically applied HDAC inhibitors and to apply them in other diseases, selective HDAC inhibitors have been developed as tools to unravel the function of individual HDAC isoenzymes, and as lead compounds in drug discovery. During recent years, HDAC inhibitors have been investigated as anticancer agents in combination therapies, and their effect on inflammatory and degenerative neurological diseases has been evaluated. 

HDAC3 has gained particular attention for its regulatory effect in cells, although further studies of the biological function of HDAC3 are still needed. Moreover, the development of selective HDAC3 inhibitors has proven to be a challenge. This is particularly attributed to the high structural similarity between the various zinc-dependent HDAC isoenzymes. As a result, a limited number of selective HDAC3 inhibitors is available, and a confined chemical space has been explored for inhibition of this particular isoenzyme. In future studies, a larger chemical space needs to be investigated, in order to find isoenzyme-selective HDAC inhibitors with favorable physiochemical properties.

## Figures and Tables

**Table 1:** Excerpt of a structural alignment of HDAC3 with HDAC 1 and 2 reveals five differences between HDAC3, and HDAC1 and 2. The amino acids are numbered according to the sequence of HDAC3.

	13	29	92	107	199
**HDAC3**	Val	Leu	Asp	Tyr	Phe
**HDAC2**	Val	Ile	Glu	Ser	Tyr
**HDAC1**	Ile	Ile	Glu	Ser	Tyr

**Table 2:** The structures of HDAC inhibitors with HDAC IC_50_ or K_i_ values in µM.

Inhibitor	Structure	HDAC1	HDAC2	HDAC3	HDAC4	HDAC5	HDAC6	HDAC7	HDAC8	HDAC10	Ref.
**Entinostat (MS-275)**	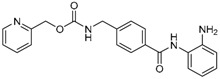	0.19	0.41	0.95	_	_	_	_	76.5	_	[19]
**RGFP966**	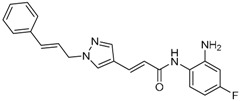	28	17	0.08	_	_	_	_	>100	_	[63]
**PD-106**	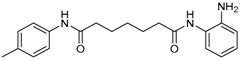	0.14 *	_	0.014 *	_	_	_	_	5 *	_	[55]
**RGFP109 (RG2833)**	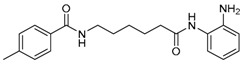	0.032 *	_	0.005 *	_	_	_	_	_	_	[57]
**BRD3308**	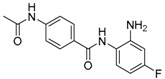	1.08	1.15	0.064	_	_	_	_	_	_	[22]

*: value is determined as Ki.

**Table 3:** The structures of novel selective HDAC3 inhibitors; IC_50_ values in µM.

Inhibitor	Structure	HDAC1	HDAC2	HDAC3	HDAC4	HDAC5	HDAC6	HDAC7	HDAC8	HDAC10	Ref.
**1**	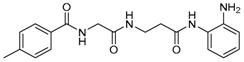	84	_	4	_	_	_	_	_	_	[58]
**2**	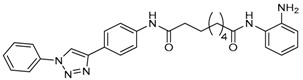	>30	>30	0.12	_	_	>30	_	>30	>30	[59]
**3**	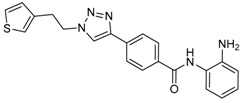	>100	19	0.24	>100	_	>100	_	>100	_	[49]
**4**	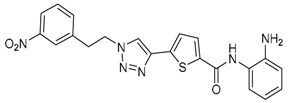	>100	>100	0.26	>100	_	>100	_	>100	_	[49]
**5**	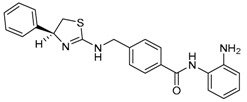	0.93	0.085	0.012	_	_	>20	_	4	_	[60]
**6**	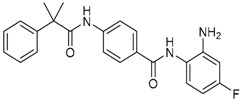	>10	>10	0.35	>10	>10	>10	>10	>10	_	[61]
**7**	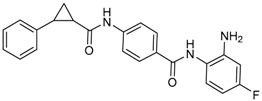	>10	>10	0.2	>10	>10	>10	>10	>5	_	[61]
**8**	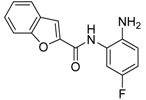	1.2	1.5	0.08	_	_	_	_	_	_	[62]
**9**	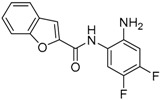	5.8	7.9	0.17	_	_	_	_	_	_	[62]
